# Methods of Control of Parasitic Weeds of the Genus *Cuscuta*—Current Status and Future Perspectives

**DOI:** 10.3390/plants14152321

**Published:** 2025-07-27

**Authors:** Lyuben Zagorchev, Tzvetelina Zagorcheva, Denitsa Teofanova, Mariela Odjakova

**Affiliations:** 1 Faculty of Biology, Sofia University “St. Kliment Ohridski”, 8 Dragan Tsankov Blvd., 1164 Sofia, Bulgaria; teofanova@biofac.uni-sofia.bg (D.T.); modjakova@biofac.uni-sofia.bg (M.O.); 2 AgroBioInstitute, Agricultural Academy, 8 Dragan Tsankov Blvd., 1164 Sofia, Bulgaria; tzvetelina.zagorcheva@gmail.com; 3 Research and Development and Innovation Consortium, Sofia Tech Park JSC, 111, Tsarigradsko Shose Blvd., 1784 Sofia, Bulgaria

**Keywords:** dodders, herbicides, allelopathy, biological control, weeds

## Abstract

Dodders (*Cuscuta* spp.; Convolvulaceae) are parasitic weeds that pose major challenges to agriculture due to their ability to infect a wide range of host plants, extract nutrients, and transmit pathogens. Their control is especially challenging because of the seed longevity, resistance to herbicides, and the capacity for vegetative regeneration. Mechanical methods such as hand-pulling or mowing are labour-intensive and often ineffective for large infestations. Chemical control is limited, as systemic herbicides often affect the host species equally, or even worse than the parasite. Current research is exploring biological control methods, including allelopathic compounds, host-specific fungal pathogens, and epiparasitic insects, though these methods remain largely experimental. An integrated approach that combines prevention, targeted mechanical removal, and biological methods offers the most promising path for long-term management. Continued research is essential to develop effective, sustainable control strategies while exploring possible beneficial uses of these complex parasitic plants. The present review aims to thoroughly summarise the existing literature, emphasising the most recent advances and discussing future perspectives.

## 1. Introduction to *Cuscuta* spp. as Invasive Weeds

*Cuscuta* spp., commonly known as dodders, is a genus of obligate parasitic plants belonging to the Convolvulaceae family. It comprises approximately 200 species in four subgenera—*Monogynella*, *Grammica*, *Cuscuta*, and *Pachystigma* [1]—differing in morphological threats, including reproductive organs, but also in the degree of plastome reduction [2]. They are characterised by thin, twining, leafless stems that coil around host plants and form haustoria—specialised structures that penetrate the host’s vascular system to extract water and nutrients. Because they lack functional roots and sufficient photosynthetic ability [3], dodders are entirely dependent on their hosts for survival [4]. Within the classification of parasitic plants, *Cuscuta* spp. are regarded as stem holoparasites—infecting above-ground host tissues with insufficient photosynthesis to support their own needs [5]. Host detection is critical in dodders’ lifecycle and is believed to be a combination of light stimuli—perception of high far red-to-red light, transmitted through the leaves of potential hosts [6] and chemical cues, e.g., volatiles, which are released by the host [7], suggesting that the parasite is able to locate and grow toward compatible, healthy plants. The invasive potential of *Cuscuta* is significant due to its broad host range, rapid growth, and capacity to spread through both seeds and vegetative fragments [8,9]. Many species, such as *Cuscuta campestris* Yunck. and *Cuscuta pentagona* Engelm., can parasitize hundreds of plant species, including important agricultural crops like alfalfa, tomato, sugar beet, and legumes [8,10,11]. Besides crop plants, *Cuscuta* are also commonly parasitizing weed species like *Polygonum aviculare* L. and *Ambrosia artemisiifolia* L [8,12]. Most of the susceptible hosts are eudicotyledonous species, while monocots are considered non-compatible [13]. However, although considered generalists, most species display preferences, determined by environmental factors [14,15,16]. Once established, *Cuscuta* can reduce host plant vigour, suppress growth, and drastically decrease yield by interfering with nutrient flow and photosynthesis [17]. Moreover, *Cuscuta* can act as a bridge for pathogens, transmitting viruses and other diseases between infected and healthy host plants via their vascular connections, which further amplifies its ecological threat [18,19].

*Cuscuta* spp. has also been investigated in pharmacological studies. Various extracts have shown antioxidant [20], anti-inflammatory [21], hepatoprotective [22], and neuroprotective [23] properties. Some studies suggest that compounds found in *Cuscuta* seeds can inhibit lipid peroxidation and protect against oxidative stress [24], making them potentially valuable in preventing or mitigating chronic diseases such as diabetes, cardiovascular conditions, and neurodegenerative disorders. Although most of these studies are preliminary and based on in vitro or animal models, they have sparked growing interest in the plant as a source of bioactive compounds. The alkaloids, flavonoids, lignans, and polysaccharides isolated from different species contribute to its diverse pharmacological profile. Several recent review papers offer an exhaustive overview of all these phytoconstituents [25,26]. Unlike other parasitic plants [27], *Cuscuta* spp. are not known to be cultivated on purpose, thus suggesting that seeds and vegetative material collected for medicinal use may represent a non-intentional way to control populations and invasion.

The genus *Cuscuta* has a cosmopolitan distribution, with species found on every continent except Antarctica [28]. In terms of native distribution, *Cuscuta* species are most diverse in tropical and subtropical regions. North and Central America, particularly Mexico and the southwestern United States, are considered major centres of diversity, hosting numerous native species, especially of the subg. *Grammica* [29]. South and Southeast Asia are rich in tropical *Cuscuta* species as well, including some of the more robust and woody types of the subg. *Grammica* and *Monogynella*. Europe, Africa, and the Middle East support fewer native species, mostly of the subgenera *Cuscuta* and *Monogynella*, while the less-studied and most recently recognised subgenus *Pachystigma* is of predominantly African distribution [30].

**Figure 1:**
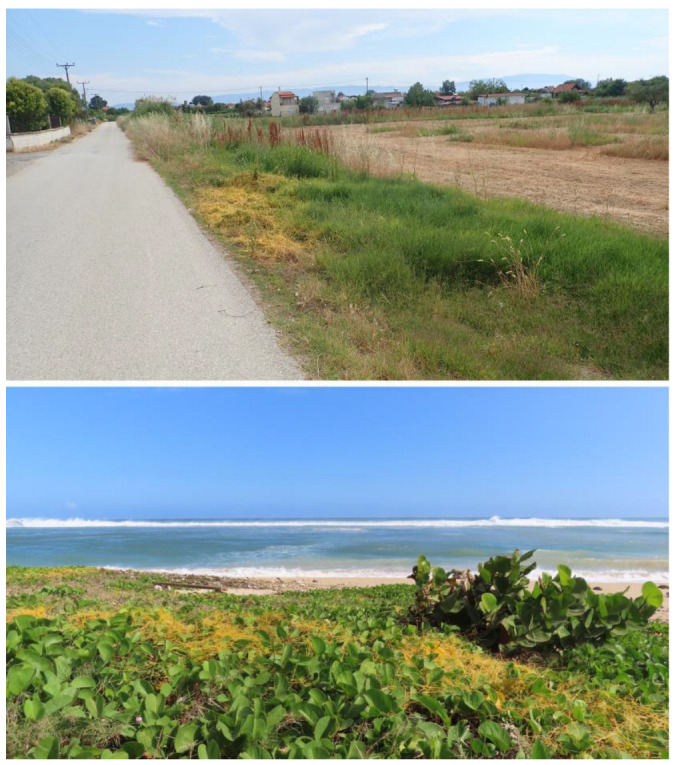
*Cuscuta campestris* infestation near the road (in Greece) and on the coastline (La Reunion Island).

Environmental factors and human activities contribute to the spread of *Cuscuta* [9]. Its seeds can remain dormant in soil for years and are often inadvertently dispersed through contaminated crop seeds, machinery, or irrigation water. Climate change and habitat disturbance further enhance its invasion by creating favourable conditions in new areas. Given its aggressive nature and potential to disrupt native ecosystems and agricultural productivity, at least several species of *Cuscuta* are considered a serious invasive threat in many parts of the world [11]. For instance, *Cuscuta campestris* (subg. *Grammica*), originally from North America, has spread extensively to Europe, Asia, Africa, South America, and Australia, often through contaminated crop seeds [11]. It is currently distributed in various environments (Figure 1). *Cuscuta japonica* Choisy (subg. *Monogynella*), native to East Asia, has invaded parts of the United States, including California, Oregon, and the southeastern states, where it parasitises woody plants [31]. Another example is *Cuscuta reflexa* Roxb., which originated in South and Southeast Asia, but has become invasive in various areas of the world [9,13]. Another species of concern is *Cuscuta europaea* L., or European dodder, which is native to Europe and Asia but has been introduced to North America [32].

Due to their wide host ranges, ability to spread through both seed and vegetative fragments, and significant impact on biodiversity and crop yields, *Cuscuta* species are listed as noxious weeds or quarantine pests in many countries [33]. Organisations such as the USDA, the European and Mediterranean Plant Protection Organisation (EPPO), and CABI monitor and regulate these species to prevent further spread and damage. According to USDA, all *Cuscuta* species except native ones are listed as federal noxious weeds (https://www.aphis.usda.gov/sites/default/files/weedlist.pdf, accessed on 26 June 2025).

Although the focus of dodders’ impact is mostly on their agroeconomic effect, they also affect natural ecosystems. The ecological impact of invasive *Cuscuta* species is profound and multifaceted, affecting both natural ecosystems and agricultural landscapes. One major ecological consequence is the loss of plant biodiversity [34,35]. By parasitizing dominant or keystone plant species, *Cuscuta* can alter community structure, reduce native species richness, and open space for secondary invasions by other alien plants. While negative within the introduced range, such a controlling effect of the parasite is often considered beneficial within the native range [16]. *Cuscuta* spp., and parasitic plants in general, can further interfere with arthropods and soil microbiota [36], whose effect might be beneficial in the native range but rather negative when introduced. It is possible, but not widely reported, that invasive *Cuscuta* species can also threaten endangered and endemic native plants.

An interesting question is whether introduced *Cuscuta* species, being similar in ecology to native ones, could outcompete them and threaten the biodiversity within the genus itself. Considering the distribution of *C. campestris* in Europe, it seems that the distribution range with native *Cuscuta* spp. rarely overlaps [18]. Other studies showed a comparatively low percentage of host plant species, shared by the introduced and native parasite—between 0.4% (*C. campestris* vs. *C. approximata* Bab.) and 22% (*C. campestris* vs. *C. europaea*) [8]—also not supporting such a hypothesis.

The current data can be summarised into several major conclusions. *Cuscuta* spp. can be considered invasive weeds, when introduced outside their native range, and there are numerous examples of such events between continents in every direction. Both introduction to new habitats and dispersal are mostly associated with human activity. When established, they have a profound effect on the plant communities and on the ecosystem overall, which is beneficial in the native range but could be negative when introduced as alien species.

## 2. Challenges to *Cuscuta* Control

The control of *Cuscuta* spp. poses several unique challenges that distinguish it from managing other weeds and even other parasitic plants. These challenges come largely from its specialised biology and parasitic lifestyle, which complicate conventional control methods. Such characteristics require an integrated weed management strategy at several stages of the plant lifecycle (Table 1), which are further explored throughout the review.

Dodders produce numerous long-lived seeds, up to 16,000 from a single plant of *C. campestris* [37]. Seeds, due to strong physical dormancy [38], can remain viable in the soil for 15 or more years [29]. This persistent seed bank increases the risk of reinfestation and means that control efforts must be sustained over multiple seasons. The small seed size facilitates accidental spread via contaminated crop seeds, farm machinery, and animal movement, complicating quarantine and sanitation efforts [33]. Such features are common in parasitic plants from distant lineages, like members of *Orobanchaceae*, in which seeds germinate only in the presence of specific compounds, released by a suitable host—strigolactones [39].

However, no chemical germination stimulants have been identified for *Cuscuta* so far. This hinders the possibility of employing the suicide germination approach in *Cuscuta* control. In this strategy, the soil seed bank of various root parasites is depleted by the preliminary application of germination stimulants in the absence of a suitable host, causing the death of the seedlings [40]. Various synthetic analogues of strigolactones, GR24 being the most famous, are continuously developed and tested for the control mainly of *Striga* spp. [41,42,43], but also *Orobanche* spp. [44] and *Phelipanche* spp. [45]. A variation in this approach is the use of trap crops, plant species which ideally trigger germination but do not serve as suitable hosts. One of many examples in root parasites is the use of maize to stimulate germination of *Orobanche cumana* Wallr., a prominent parasite on sunflower [46]. Several monocots were reported as possible trap crops in the management of *Cuscuta* spp. [47], as germinated seedlings attach to them and die out due to host–parasite incompatibility. Crop rotation with cereals is often applied as a means of control although seed longevity does not allow for complete removal [48]. However, such an approach is obviously less effective, as it may eliminate already germinated seedlings without causing additional germination as in phytochemical-driven germination in root parasites.

When already attached, the multiple haustorial connections mean that herbicides or mechanical removal must either target the parasite without damaging the host or be applied very early, before attachment occurs. Physical cutting or hand-pulling often leaves behind fragments capable of propagating, while the removal of dodder can be labour-intensive and may damage the host [48]. Additionally, the wide host range of many *Cuscuta* species, which can parasitize hundreds of plant species—including crops, ornamentals, and native plants—makes crop rotation and host removal less effective as standalone strategies. This broad host adaptability allows *Cuscuta* to survive and spread in diverse environments.

## 3. Mechanical Control

The management of dodder infestation could be more effective when proper prevention is applied. Mechanical techniques for dodder removal are the primary means of parasitic weed management before and after *Cuscuta* emergence. They could be applied on three different stages—to prevent seed contamination, to reduce the soil seed bank, and after infestation, when dodder plants are already established.

The most common means of spreading is by distribution of seeds within commercial packages of crop seeds (most commonly alfalfa), contaminated agricultural equipment, and animal feeding [48]. Therefore, sanitation measures could significantly reduce the possibility of the unintentional introduction of *Cuscuta* seeds into new areas. The most obvious approach is to avoid the collection of crop seeds from *Cuscuta*-infested fields. Obviously, this is a suitable approach, but it does not entirely exclude the possibility of contaminating the seed packaging. Further treatment of commercial seed packages could significantly limit the spread. Several approaches were published recently, including dry-heat treatment up to 120 °C, which was shown to significantly reduce *Cuscuta japonica* and *Cuscuta pentagona* seed viability while not affecting crop seeds [49]. Heat treatment is also essential in devitalizing *Cuscuta* seeds in compost manure, although *C. campestris* was shown to be far more resistant than *Phelipanche aegyptiaca* Pers., for example [50]. Another possibility is the application of magnetic drum separation on an industrial scale, specifically suitable for the separation of dodder from alfalfa seeds [51,52]. However, variability of *Cuscuta* seed size could significantly affect the effectiveness of the process [52].

Mechanical control is a traditional and often necessary method for managing *Cuscuta* infestations, especially in situations where chemical or biological control options are limited or undesirable. Because *Cuscuta* is a parasitic plant that attaches tightly to its host, physically removing it requires careful timing and labour-intensive effort. Despite these challenges, mechanical control remains an important tool in integrated management strategies aimed at reducing dodder populations and minimising crop damage [48,53].

Before germination, as *Cuscuta* spp. germinate close to, or on the surface of the soil [33], tillage and deep ploughing may be beneficial to reduce dodder germination [48]. Once established, a common mechanical method is hand-pulling or cutting of *Cuscuta* vines from infested plants. This approach is most effective when infestations are detected early and the parasite has not yet extensively colonised the host. By cutting the dodder vines close to the host stem before they flower and set seed, the spread of new seeds can be reduced, limiting future infestations. However, because *Cuscuta* vines can regrow from small fragments, thorough removal of all parasite tissue is essential. Incomplete removal may allow the dodder to re-establish quickly, so this method often requires repeated efforts throughout the growing season.

Mechanical control methods face several challenges. The intensive labour required for hand removal can be prohibitive in large-scale agriculture, and mechanical damage to crops during dodder removal can reduce yields. Additionally, because *Cuscuta* seeds are often widely dispersed and can remain viable for many years, mechanical methods must be part of a long-term management plan to achieve meaningful reductions in infestation levels. Furthermore, timing is critical. Removing *Cuscuta* after seed production has occurred is ineffective in controlling spread, and early detection is essential for mechanical control to succeed. Monitoring fields regularly to identify and remove dodder before flowering is therefore a key component of this approach.

## 4. Chemical Methods of Control

### 4.1. Herbicides

Due to its obligate parasitic lifestyle and intimate connection with host plants, controlling *Cuscuta* with herbicides is particularly challenging. The plant’s lack of true roots and its reliance on host vascular systems mean that most systemic herbicides must affect the parasite without causing unacceptable damage to the host. Nonetheless, chemical control remains an important component of integrated *Cuscuta* management, especially in agricultural systems [48,54]. An overview of the most commonly used herbicides is provided in Table 2.

The success of herbicide-based control largely depends on the timing of application. Pre-emergence herbicides, applied before *Cuscuta* seeds germinate, are often the most effective option because they prevent the parasite from establishing contact with host plants [48]. Common pre-emergence herbicides include dinitroaniline compounds like trifluralin and pendimethalin, which inhibit cell division, are effective in suppressing *Cuscuta* germination in crops such as alfalfa, clover, and chickpea [61,62,63]. These herbicides can be incorporated into the soil or applied as surface treatments, offering control before the parasite has a chance to attach and form haustoria.

Post-emergence control, on the other hand, presents greater difficulty. Once *Cuscuta* is attached to a host, systemic herbicides applied to the host plant can translocate into the parasite but may also harm the crop. Herbicides like glyphosate, 2,4-D, and glufosinate have been tested with varying levels of success. Glyphosate, a broad-spectrum systemic herbicide, is particularly effective at killing dodder but is non-selective and often damages or kills the host plant [59,64]. Therefore, it is mostly used in non-crop areas or as a spot treatment in fallow fields and along roadsides.

Some selective post-emergence herbicides have shown promise for dodder control without severe crop injury. For instance, imazethapyr, an ALS-inhibiting herbicide, is used in legumes like alfalfa and soybean and has shown efficacy against dodder when applied early in the parasite’s development [56,65]. Similarly, clopyralid and rimsulfuron have been used to suppress dodder growth in certain crop systems [66]. The key to effective use is to apply these herbicides shortly after dodder emergence but before it penetrates host tissues.

Another approach involves host plant resistance to herbicides. Genetically modified or naturally tolerant crop varieties allow for the use of non-selective herbicides without harming the host [54]. For example, glyphosate-resistant crops (such as transgenic soybean or maize) enable the application of glyphosate post-emergence, effectively killing *Cuscuta* without damaging the crop.

Despite the range of chemical tools available, herbicide-based control of *Cuscuta* is rarely sufficient on its own. The parasite’s persistent seed bank, rapid growth, and close association with host plants make it difficult to eradicate. Moreover, overreliance on herbicides can lead to the development of herbicide-resistant weed populations and environmental concerns, such as contamination of water sources and non-target plant injury.

### 4.2. Allelopathy

Allelopathy, the biological phenomenon where plants release chemical compounds that influence the growth, survival, or reproduction of other plants, has gained attention as a promising, environmentally friendly approach to controlling parasitic weeds such as *Cuscuta* [67]. Since dodders rely heavily on host plants to survive, interfering with its germination, attachment, or growth through allelochemicals could reduce its impact without the drawbacks of synthetic herbicides.

Research into allelopathic control of *Cuscuta* focuses on identifying plants that produce natural substances capable of inhibiting dodder seed germination or preventing the parasite from successfully attaching to host plants [68]. Several plant species, including certain cover crops, weeds, and medicinal plants, are known to exude bioactive compounds into the soil or release volatile chemicals that can affect *Cuscuta*’s development. Some notable examples are presented in Table 3. Apparently, the diversity of compounds with inhibitory allelopathic effect is substantial.

One potential mechanism by which allelopathy can control *Cuscuta* is by disrupting seed germination. For example, extracts from plants like lavender [74] and sunflower [75] have shown inhibitory effects on dodder seed germination in laboratory and greenhouse studies, but also on seedlings’ growth. These findings suggest that incorporating allelopathic cover crops into crop rotations or using plant residues as soil amendments could help suppress *Cuscuta* populations.

Despite its promise, allelopathic control of *Cuscuta* faces several challenges. Overall, the effect is significant during germination and early seedling growth (pre-attachment), but negligible in infested fields. The effectiveness of allelochemicals can vary widely depending on the environmental conditions, soil type, and plant species involved [76]. The concentration and persistence of allelochemicals in the field are often lower than in controlled laboratory settings, which can limit practical application [77]. Moreover, there is a risk that allelochemicals might negatively affect crop plants or beneficial soil organisms if not carefully managed. One promising approach, demonstrated in certain studies [72], includes the identification of plants resistant to *Cuscuta* parasitism, followed by the validation of the allelopathic activity of the plant extract and further identification of the particular compounds responsive to this effect.

Allelopathy also seems to be a bi-directional interaction between parasite and host, as many *Cuscuta* species were also shown to exhibit allelopathic effects against other plant species. For example, extracts of *C. campestris* were found to inhibit the germination of radish and lettuce [78], and extracts of *C. chinensis* of were also found to inhibit the germination of chicory and alfalfa [79] and tomato [80]. The major phytochemicals responsible for this effect were caffeic acid, hydrocinnamic acid, cinnamic acid, p-coumaric acid, kaempferol, and quercetin [80].

## 5. Biological Control

Biological control of *Cuscuta* species has emerged as a promising strategy in the management of this parasitic plant, which poses a serious threat to a wide range of agricultural crops worldwide. Given its parasitic nature and lack of roots or leaves, traditional weed control methods such as mechanical removal and herbicide application often prove ineffective or impractical. This has spurred increased interest in the development and implementation of biological control methods, which offer more sustainable and environmentally friendly solutions.

Biological control of *Cuscuta* can be approached through the use of natural enemies, including insects, fungi, and other microorganisms that can specifically target and suppress dodder populations without harming host crops. One of the most studied and potentially effective agents is the fungus *Alternaria destruens E.G. Simmons*, which has demonstrated strong pathogenicity against *Cuscuta pentagona* [81]. This fungus infects dodder stems and tissues, leading to necrosis, collapse of haustoria, and eventual death of the parasite. Field and greenhouse studies have shown that *Alternaria destruens* can significantly reduce *Cuscuta* biomass and limit its spread, making it a promising candidate for large-scale biocontrol applications. Another *Alternaria*, *Alternaria alternata* (Fr.) Keissl., was also proposed as a bioherbicide for the control of *Cuscuta japonica* [82]. Commercialised bioherbicides, based on fungi for use against *Cuscuta*, include Smolder (*Alternaria destruens*-based) and Luboa-2 (*Colletotrichum gloesporiodes* f. sp *cuscutae*-based), which were reviewed previously [83]. However, as in most biocontrol agents, such efforts must be accompanied by a broad study of the phytopathogenicity of the fungi against other non-target plants, e.g., the phytopathogenic strain must be specific to the target weed [83]. In all the mentioned cases, some promising results were reported on this matter.

In addition to fungal pathogens, some insect species have been explored as biological control agents. For instance, *Melanagromyza cuscutae* Hering, 1958 lays its eggs on *Cuscuta* stems, and the larvae bore into the tissues, disrupting the parasite’s vascular system and diminishing its ability to maintain connections with the host [84,85]. Gall-forming weevils of the genus *Smicronyx* were also proposed as an epiparasitic means of control of *Cuscuta* reproductive potential [86]. However, the specificity and ecological impact of insect-based control require careful assessment to ensure that non-target plant species are not adversely affected.

Despite the good perspectives of biological control, several challenges remain. The specificity and efficacy of biocontrol agents under varying environmental conditions must be validated through extensive field trials. Regulatory frameworks governing the release of biological agents also require rigorous testing for safety and ecological impact. Moreover, integration with other management practices is crucial for long-term success, as no single method is likely to provide complete control of *Cuscuta* across all cropping systems.

## 6. Combating Invasive Alien Species

Ironically, being parasitic weeds themselves, dodders were shown to be effective in suppressing other weeds, thus limiting biological invasions. The recent data in this direction are summarised in Table 4.

Several important points must be addressed in the presented data. First, all the reports come exclusively from China, maybe because dodders are important medicinal plants in traditional Chinese medicine [97] and therefore the attitude towards them is not entirely negative. This is complemented by the traditionally strong scientific interest in controlling invasive weeds in this country. Second, introduced species like *C. campestris* and *C. gronovii* are equally effective as native dodders, which may be explained by the similarity in ecological preferences with the closely related *C. australis*, but also the fact that some of these species are well-established in native flora and well-adapted to the local environment. The most important point, however, is why local dodders display apparent preference towards alien host species. One possible hypothesis is based on the invasive features of the introduced species—fast growth and high nutrient content, which makes them a perfect target [87]. However, we are also tempted to hypothesise that local species, due to continuous parasitic pressure, have developed a certain degree of resistance to *Cuscuta*, therefore making the alien species a much easier target. Whatever the reason, there is strong evidence that native *Cuscuta* species could successfully control the spreading of invasive alien species in certain regions.

## 7. Conclusions and Future Perspectives

Due to their parasitic lifestyle, dodders were proved to be particularly difficult for agricultural and environmental management, and integrated weed management was proposed as an appropriate solution more than thirty years ago [98]. These usually include pre-emergence treatments with herbicides, followed by mechanical removal after emergence and flaming post-production to destroy the seeds of the parasite [98,99,100]. However, most of the studies are directed towards agricultural lands, while the invasion of dodders in natural environments is less studied, and usually no management strategies exist. It seems that most of the introduced species are already well-established worldwide and difficult or impossible to eradicate. In order to prevent new invasions, phytosanitary control and prevention seems to be the most important measure. While eradication strategies in agricultural lands are clearly necessary to prevent economical losses, the situation in natural environments should be carefully assessed, as dodders appeared to be important elements of plant communities. Furthermore, dodders may also represent a valuable source of phytochemicals with a variety of applications.

## Figures and Tables

**Table 1:** Measures for dodder control, needed at different stages of its lifecycle.

Life Stage	Measures	Obstacles
Seed spreading	Decontamination of commercial crop seeds and soil—mechanical methods	Small seeds, similar to many crop plant seeds
Soil seed bank	Reduction in soil seed bank—mechanical methods, crop rotation	Seed longevity and strong dormancy, broad host range
Germination and pre-attachment	Inhibition of germination—herbicides, allelochemicals, biological methods	No specific germination stimulants
Parasitism (post-attachment)	Removal of the parasite—herbicides, mechanical methods, biological methods	Strong connection to the host, vegetative propagation from small stem fragments
Reproduction	Decrease in seed production—mechanical methods, biological methods	Large quantity of seed production

**Table 2:** Non-exhaustive examples of herbicide (according to HRAC classification) treatment of *Cuscuta* spp., published between 2020 and 2025.

Herbicide	Parasite/Host Species	Time of Application	Maximum Efficacy	References
Group 2 (ALS inhibitors)				
Rimsulfuron	*Cuscuta campestris*/eggplant	Attachment	45.8% biomass reduction	[55]
Group 3 (Inhibitors of microtubule assembly)				
Propyzamide	*Cuscuta campestris*/alfalfa	Attachment	100%	[56]
Propizamide + ethofumasate **	*Cuscuta campestris/sugarbeet*	Post-emergence	100%	[57]
Pendimethalin	*Cuscuta campestris*/eggplant	Attachment	47.2% biomass reduction	[55]
	*Cuscuta campestris*/*Trifolium alexandrinum* L.	Pre- and post-emergence	83% *	[58]
Group 9 (Inhibitors of enolpyruvyl shikimate phosphate synthase)				
Imazethapyr	*Cuscuta campestris*/alfalfa	Attachment	96%	[56]
	*Cuscuta campestris*/*Trifolium alexandrinum* L.	Pre- and post-emergence	80%	[58]
Glyphosate	*Cuscuta campestris*/alfalfa	Attachment	82% *	[56]
		Attachment	97.5%	[59]
	*Cuscuta campestris*/*Nerium oleander* L.	Established	95%	[60]
Group 14 (Inhibitors of protoporphyrinogen synthase)				
Oxyfluorfen	*Cuscuta campestris*/*Trifolium alexandrinum* L.	Post-emergence	73% *	[58]

* With strong negative effect on host crop plant; ** Ethofumasate is group 16.

**Table 3:** Examples of plant extracts with allelopathic inhibitory effects on *Cuscuta campestris*.

Plant Species	Compounds	Effect	References
Isolated compounds	2-benzoxazolinone, hydrocinnamic acid, pisatin	decreased germination, seedling necrosis	[69]
Isolated compounds	analogues of hydrocinnamic acid	seedling necrosis	[70]
*Rhazya stricta* Decne.	not identified—aqueous leaf extract	decreased germination and seedling growth	[71]
*Conyza bonariensis* (L.) Cronq.	(4Z)-lachnophyllum lactone	decreased seedling growth	[72]
*Nepeta meyeri* Benth.	not identified—aqueous leaf extract	decreased germination and seedling growth	[73]
*Lavandula angustifolia* Mill.	not identified—aqueous leaf extract	decreased germination	[74]

**Table 4:** Reports on *Cuscuta* spp. limiting the development of invasive alien plants.

Cuscuta Species	Affected Invasive Species	Location	Reference
*Cuscuta australis* R.Br. (N)	*Ipomoea cairica* (L.) Sweet, *Mikania micrantha* Kunth, and *Wedelia trilobata* (L.) Pruski	China	[87]
*Cuscuta campestris* (I)	*Mikania micrantha*	China	[88,89,90]
*Cuscuta australis* (N)	*Xanthium italicum* Moretti	China	[91]
*Cuscuta japonica* (N)	*Ambrosia trifida* L.	China	[92]
*Cuscuta gronovii* Willd. (I)	*Celosia argentea* L., *Sphagneticola trilobata* (L.) Pruski, *Crotalaria pallida* Aiton	China	[93]
*Cuscuta australis* (N)	*Humulus scandens* (Lour.) Grudz.	China	[15]
*Cuscuta campestris* (I)	*Ipomoea purpurea* (L.) Roth	China	[94]
*Cuscuta australis* (N)	*Mikania micrantha*	China	[95]
*Cuscuta campestris* (I)	*Solanum rostratum* Dunal	China	[96]

N—native; I—introduced.

## Abbreviations

The following abbreviations are used in this manuscript:

ALS	Acetolactate Synthase
CABI	Commonwealth Agricultural Bureaux International
EPPO	European and Mediterranean Plant Protection Organisation
GR24	Synthetic Strigolacton Analogue
HRAC	Herbicide Resistance Action Committee
USDA	United States Department of Agriculture
